# Revisitingmolecular serotyping of *Streptococcus pneumoniae*

**DOI:** 10.1186/1471-2164-16-S5-S1

**Published:** 2015-05-26

**Authors:** Dhian RA Camargo, Fabiano S Pais, Ângela C Volpini, Marluce AA Oliveira, Roney S Coimbra

**Affiliations:** 1 Biosystems Informatics, Research Center René Rachou (CPqRR), Oswaldo Cruz Foundation (FIOCRUZ), Belo Horizonte, MG, 30190-002, Brazil; 2 Genomics and Computational Biology Group, CPqRR, FIOCRUZ, Belo Horizonte, MG, 30190-002, Brazil; 3 Center for Excellence in Bioinformatics, CPqRR, FIOCRUZ, Belo Horizonte, MG, 30190-002, Brazil; 4 Service of Bacterial and Fungal Diseases, Ezequiel Dias Foundation (FUNED), Belo Horizonte, MG, 30510-010, Brazil

**Keywords:** *Streptococcus pneumoniae*, *cps *gene cluster, molecular serotyping, typing methods, *cps*-RFLP, Bioinformatics

## Abstract

**Background:**

Ninety-two *Streptococcus pneumoniae *serotypes have been described so far, but the pneumococcal conjugate vaccine introduced in the Brazilian basic vaccination schedule in 2010 covers only the ten most prevalent in the country. Pneumococcal serotype-shifting after massive immunization is a major concern and monitoring this phenomenon requires efficient and accessible serotyping methods. Pneumococcal serotyping based on antisera produced in animals is laborious and restricted to a few reference laboratories. Alternatively, molecular serotyping methods assess polymorphisms in the *cps *gene cluster, which encodes key enzymes for capsular polysaccharides synthesis in pneumococci. In one such approach, *cps*-RFLP, the PCR amplified *cps *loci are digested with an endonuclease, generating serotype-specific fingerprints on agarose gel electrophoresis.

**Methods:**

In this work, *in silico *and *in vitro *approaches were combined to demonstrate that XhoII is the most discriminating endonuclease for *cps*-RFLP, and to build a database of serotype-specific fingerprints that accommodates the genetic diversity within the *cps *locus of 92 known pneumococci serotypes.

**Results:**

The expected specificity of *cps*-RFLP using XhoII was 76% for serotyping and 100% for serogrouping. The database of *cps*-RFLP fingerprints was integrated to Molecular Serotyping Tool (MST), a previously published web-based software for molecular serotyping. In addition, 43 isolates representing 29 serotypes prevalent in the state of Minas Gerais, Brazil, from 2007 to 2013, were examined *in vitro*; 11 serotypes (nine serogroups) matched the respective *in silico *patterns calculated for reference strains. The remaining experimental patterns, despite their resemblance to their expected *in silico *patterns, did not reach the threshold of similarity score to be considered a match and were then added to the database.

**Conclusion:**

The *cps*-RFLP method with XhoII outperformed the antisera-based and other molecular serotyping methods in regard of the expected specificity. In order to accommodate the genetic variability of the pneumococci *cps *loci, the database of *cps*-RFLP patterns will be progressively expanded to include new variant *in vitro *patterns. The *cps*-RFLP method with endonuclease XhoII coupled with MST for computer-assisted interpretation of results may represent a relevant contribution to the real time detection of changes in regional pneumococci population diversity in response to mass immunization programs.

## Background

*Streptococcus pneumoniae *is a Gram-positive coccus, with more than 90 serotypes, and is one of the most important agents of pneumonia, meningitis and sepsis in children worldwide [1,2]. In Brazil, between 2004 and 2006, pneumococcal disease was responsible for 34,217 hospitalizations in the Brazilian Unified Health System (0.1% of the total number of hospitalizations), and pneumonia represented 64.8% of this total. Pneumococci also caused 31.3% of all confirmed cases of bacterial meningitis [3].

Pneumococcal disease can be prevented by vaccination. In 2010, the 10-valent pneumococcal conjugate vaccine PCV10, covering serotypes 1, 4, 5, 7F, 6B, 9V, 14, 18C, 19F, and 23F, was introduced in the Brazilian basic vaccination schedule. It is reasonable to expect that the prevalence of pneumococcal diseases in this country will be drastically reduced within a few years. However, pneumococcal serotype-shifting after massive immunization is a major concern and monitoring this phenomenon requires efficient and accessible serotyping techniques [4,5].

The Quellung reaction is the gold standard method for pneumococci serotyping. This method relies on the recognition of capsular polysaccharides (CPS) by serotype or serogroup-specific antibodies. The current serotyping scheme requires a full set of expensive antisera prepared in animals, is laborious, and error-prone due to cross-reactivity between some CPS [6,7]. Due to the large number of anti-sera needed for complete serotyping of bacterial isolates, laboratories with the complete panel of anti-sera are scarce. In Brazil, the Adolf Lutz Institute is the only reference center for serotyping.

Pneumococcal CPS is generally synthesized by the Wzx/Wzy-dependent pathway. The enzymes responsible for CPS synthesis are encoded by a set of genes located at the CPS biosynthetic (*cps*) loci, which is flanked by conserved genes *dexB *and *aliA*. Exceptions are serotypes 3 and 37 that use the synthase pathway [8,9].

An alternative molecular serotyping method for *Shigella *spp. and *Escherichia coli *has already been published [10,11]. The method named *rfb*-RFLP relies on restriction fragment length polymorphisms (RFLP) of the *rfb *loci, responsible for the synthesis of the somatic antigen in *E. coli *and *Shigella *spp. A database with *rfb*-RFLP patterns of all known serogroups/serotypes of this genospecies has been published, and a web-based software has been developed to compare the *rfb*-RFLP patterns of clinical isolates with those in the database [12]. This technique has been successfully used for more than a decade and allowed the discovery of new putative serotypes [13-15].

We present herein a new tool for *S. pneumoniae *molecular serotyping based on RFLP of the PCR-amplified *cps *locus (*cps*-RFLP). This tool includes: 1) a Molecular Biology method, which produces serotype-specific fingerprints; 2) a database containing the reference fingerprints; 3) a software to predict the serotype of clinical samples by comparing their fingerprints with those in the reference database.

## Methods

All reagents were manufactured by Sigma-Aldrich (Saint Louis, MO), except when indicated in the text.

### Bioinformatics analysis

One hundred and seven sequences of *cps *loci representing 92 serotypes were downloaded from GenBank (http://www.ncbi.nlm.nih.gov/Genbank) (Table S1, Additional file 1) [9,16-18].When two or more sequences where available for a given serotype, all *cps *loci sequences were analysed to assess the diversity within serotype.

The *cps *sequences were screened for internal endonuclease cleavage sites using REMAP, from European Molecular Biology Open Software Suite (EMBOSS) (http://emboss.sourceforge.net). Enzymes with four to 26 restriction sites at each *cps *locus were selected for further analysis. For each selected enzyme, a database was built with *in silico *restriction patterns for each serotype generated with RESTRICT, also from EMBOSS package. This *in silico *analytical pipeline was applied also to the endonuclease HinfI, which had already been proposed for molecular serotyping of a subset of pneumococcal serotypes [19]. The most discriminant enzyme was chosen using a standalone version of Molecular Serotyping Tool (MST), our previously published software for computer-assisted molecular serotyping [12]. Briefly, pairwise alignments of all *cps*-RFLP patterns were performed and the pairwise distances were calculated as the sum of the penalties for the edit operations required to transform one pattern to the other. The most discriminant enzyme returned the highest median value for the calculated distances between all pairs of *cps*-RFLP patterns and the lowest number of indistinguishable pairs.

The NEIGHBOR program of Phylogeny Inference Package (PHYLIP) (http://evolution.genetics.washington.edu/phylip.html), with Unweighted Pair Group Method with Arithmetic Mean (UPGMA) as the linkage method, was used to cluster the *cps*-RFLP patterns based on the distance matrices produced by MST software. The dendrograms were visualized and edited with FigTree (http://tree.bio.ed.ac.uk/software/figtree/).

### Bacteria isolate serotyping

Forty-five clinical isolates of *S. pneumoniae *representing 31 serotypes (Table 1) isolated at the CentralPublic Health Laboratory (FUNED) of the state of Minas Gerais, Brazil, from 2007 to 2013 and ATCC49619 reference strain (serotype 19F) were analysed. The 31 serotypes correspond to 83.7% of prevalent pneumococcal serotypes in children younger than five years in Brazil [20].

**Table 1:** *Streptococcus pneumoniae *isolates tested *in vitro*.

Strain designation	Serotype	*cps*-RFLP pattern
295/08-LP	1	E_1
381/11-LCR, 159/11-LCR	3	- *
345/11-LCR	4	E_4
197/11-HEM	5	E_5
103/11-LCR, 305/08-HEM, 262/08-LCR, 619/08-LCR	6A	E_6A
143/11-LCR	6B	E_6B
127/10-LCR, 387/11-LCR	6C	E_6C
176/08-LCR, 648/10-LCR	7C	E_7C
149/11-LCR, 620/09-LCR	9N	E_9N
585/10-LCR, 511/11-LCR	9V	E_9V
421/10-LCR, 490/11-LCR	10A	E_10A
084/11-LCR	12F	E_12F
148/10-LCR	13	E_13
198/11-HEM, 380/12-LCR	14	E_14
779/09-LCR	15C	E_15C
156/11-LCR	16F	E_16F
595/11-LCR	17F	E_17F
586/10-LCR	18A	E_18A
240/11-LCR	18B	E_18B
120/11-LCR	18C	E_18C
124/11-LCR, 649/11-LP	19A	E_19A
254/11-LCR, 24/12-LCR	19F	E_19F
ATCC49619	19F	E_19F'
509/12-LCR	19F	E_19F''
443/10-LCR	22F	- *
159/09-LCR	23B	E_23B
029/11-LCR	23F	E_23F
080/11-LCR	24F	E_24F
435/09-LCR	28A	E_28A
79/11-HEM	29	E_29
317/08-LCR	34	E_34
144/09-LCR, 192/11-LCR	35B	E_35B
169/11-HEM	35F	E_35F

* PCR amplification of *cps *loci failed for these isolates.

Pneumococci identification was confirmed by Gram stain, colonial morphology on blood agar, susceptibility to optochin (5 micrograms disks), and bile solubility [7]. All isolates were serotyped by Quellung reaction [7,21] at Adolfo Lutz Institute, Brazilian Reference Laboratory for Bacterial Meningitis, using specific antisera (Statens Serum Institut, Copenhagen, Denmark).

### Genomic DNA preparation

*S. pneumoniae *strains were grown overnight on blood agar and the DNA was extracted using the method originally described by Coimbra *et al *[10] and adapted to *S. pneumoniae*. Briefly, colonies were harvested from agar and resuspended in sterile saline. Cell titer was estimated by measuring the optical density in turbidimeter (Biomèrieux, Marcy l'Etoile, France). A volume containing approximately 1.8 × 10^8 ^bacteria was centrifuged at 3000 *g *for 15 minutes (min) at 4°C and pellets were resuspended in 10 ml of washing buffer (1 M NaCl; 10 mM Tris-HCl; pH 7.6). After a second centrifugation step, the pellet was resuspended in 50 μl of washing buffer, 15 μl of lysozyme at 20 mg/ml and 3 μl of the mutanolysin at 5 U/ml. Sixty-eight microliters of 2% low-melting-point agarose prepared with TE buffer (10 mM Tris-HCl; 1 mM EDTA; pH 8.0) was added to the mix. The mixture was homogenized and incubated at 41°C for 10 min. Then, aliquots of 20 μl were pippeted onto glass slides covered with Parafilm and let to solidify. Plugs were then transferred into 15 ml Falcon tubes (Bacton Dickinson Labware, Franklin Lakes, NJ) containing 1 ml of lysis buffer (1 M NaCl; 100 mM EDTA; 6 mM Tris-HCl; 0.25% Brij 58; 0.2% deoxycholate; 0.5% *N*-lauroylsarcosine, [pH 8.0]) supplemented with 5 μl of mutanolysin (5 U/ml), 50 μl of lysozyme (20 mg/ml) and 10 μl of RNase I (50 mg/ml) and tubes were incubated at 37°C overnight. After that, the lysis buffer was discarded, 1 ml of ES buffer (0.5 M EDTA, pH 9,1% N-lauryl sarcosyl) containing 0.1 mg/ml of proteinase K was added, and tubes were incubated at 51°C overnight. Then, ES buffer was discarded and plugs were washed six times in 10 ml of 1X TE for 60 min at room temperature. One plug (approximately 20 μl) was melted at 68°C for 15 min in 20 μl of 1X TE and 3 μl were used as DNA templates for the PCR reaction. The DNA was evaluated for quality and quantity by electrophoresis in 0.6% agarose gels, with TBE buffer (89 mM Tris-base; 89 mM boric acid; 2.5 mM EDTA; pH 8.0), at 4.5 V/cm between electrodes for 90 min. The fragments sizes were roughly estimated using the lambda Hind III ladder (Promega, Madison, WI) and the GelAnalyzer software (http://www.gelanalyzer.com). This extraction method was chosen because it yields large and high-integrity DNA fragments suitable for long-distance PCR [10,11].

### PCR *cps *amplification

Oligonucleotides DexB2 (5'-GAC CGT CGC TTC CTA GTT GT-3') and AliA2 (5'-ATG CAG CTA AAG TAG TCG CC-3'), respectively complementary to *dexB *and *aliA*[19], were used to amplify the *cps *gene clusters. Amplification was performed using AccuTaq LA DNA polymerase. Three microliters of template DNA was added to the amplification solution containing 0.5 μl Taq (2.5 U), 5 μl buffer, 1 μl DMSO, deoxynucleoside triphosphates at 0.5 mM, and 0.6 mM of primers in a final volume of 50 μl. Cycling conditions were programmed as follows: one denaturation step at 93°C for 2 min and 10 initial cycles of 93°C for 15 seconds (sec), 50°C for 30 sec, and 68°C for 20 min, followed by 25 iterative cycles of 93°C for 15 sec, 50°C for 30 sec, and 68°C for 20 min plus 15 sec for each new cycle. A final elongation step of 68°C for 10 min was run. Amplicons were verified by electrophoresis as above. PCR product sizes were estimated using the lambda *Hin*d III DNA ladder (Promega, Madison, WI) and the GelAnalyzer software.

### *cps*-RFLP

Twenty-five microliters of each amplified product were digested using XhoII as follows. PCR products were incubated with 1 μl enzyme (10 U), 5 μl digestion buffer (Promega, Madison, WI) for 4 hours (h) at 37°C according to the manufacturer's instructions. Restriction fragments were separated by electrophoresis in 20 × 25 cm gels made of 1.5% ultrapure agarose (Invitrogen, Carlsbad, CA) in 0.5X TAE buffer (20 mM Tris-acetate; 0.5 mM EDTA; pH 8.0) at 4.5 V/cm between electrodes for 4 h. Standard molecular weight GeneRuler 1 kb Plus DNA Ladder (Thermo Scientific, Wilmington, DE) was selected as molecular marker. After electrophoresis, gels were stained for 45 min with 0.5 μg/ml ethidium bromide and destained twice for 15 min in distilled water. Gel images were electronically captured using a charge-coupled device (CCD) video camera interfaced to a microcomputer. Tagged image file format (TIFF) images were collected and the molecular weights of fragments were estimated using the GelAnalyzer, with the following parameters: Rolling ball: 25, MW calibration: Log fit. Bands corresponding to fragments smaller than 250 and larger than 4,300 bp were not considered because fragments sizing above and below these thresholds are more error-prone [10,11].

### Reference database

The *cps*-RFLP patterns obtained *in silico *were uploaded to the database of the web-based MST software [12], which is freely accessible at http://www.cebio.org/mst. In addition, the database was complemented with the *in vitro *patterns that did not match any of the *in silico *ones.

### Statistical analysis

The ensembles of MST distances between all pairs of *cps*-RFLP patterns predicted *in silico *for BslFI, Eco57MI, HindII, HinfI, and StyI were compared to the ensemble predicted for XhoII using Friedman test followed by Dunn's Multiple Comparison Test. Differences were considered significant when p < 0.05. Statistical analyses were performed using GraphPad Prism 5.02 (GraphPad Software Inc, San Diego, CA).

## Results

### Bioinformatics analysis

**Figure 1:**
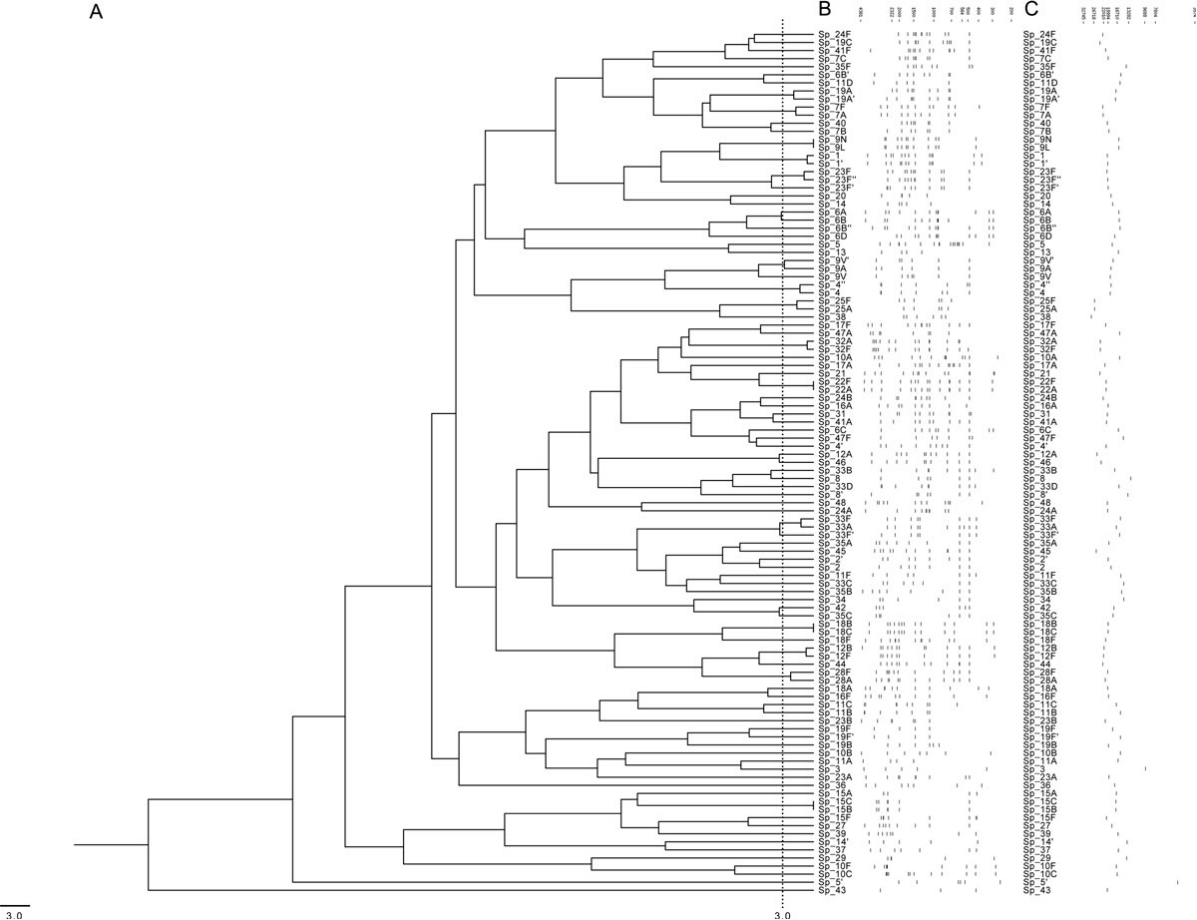
**Clustering the *in silico cps*-RFLP patterns calculated for the reference strains**. (**A**) Dendrogram showing the results of clustering the 107 *cps*-RFLP patterns generated by *in silico *restriction with XhoII endonuclease. The dashed line represents the distance threshold under which patterns are indistinguishable by MST software; (**B**) Schematic representation of the *cps*-RFLP patterns; and (**C**) their respective *cps *amplicons. Fragment sizes are in base pairs.

*In silico *restriction analysis disclosed five candidate endonucleases. For each of these enzymes, namely BslFI, Eco57MI, HindII, StyI and XhoII, a database was built with the restriction patterns calculated for each serotype. These *cps*-RFLP patterns were represented by strings of comma-separated, size-ordered fragments (within the thresholds from 250 to 4,300 bp). For each endonuclease, MST aligned all pairs of *cps*-RFLP patterns producing a distance matrix from which the median, mean, standard deviation and the number of indistinguishable serotype pairs under the selected threshold of 3.0 for MST distance were calculated. XhoII was the most discriminating endonuclease with the highest median distance between pairs of *cps*-RFLP patterns and the lowest number of indistinguishable serotype pairs (Table 2). The *cps*-RFLP patterns predicted for XhoII consist of three to 17 restriction fragments ranging from 254 to 4,274 bp (Figure 1). MST clearly distinguished 70 *in silico *serotype-specific *cps*-RFLP patterns obtained with XhoII. However, the following pairs of serotypes were indistinguishable: 7A/7F; 9A/9V; 9L/9N; 12B/12F; 15B/15C; 18B/18C; 22A/22F; 25A/25F; 28A/28F; 32A/32F and 33A/33F. Thus, the expected specificity was 76% for serotyping and 100% for serogrouping. For HinfI, the *in silico cps*-RFLP patterns had nine to 44 restriction fragments ranging from 250 to 4,120 bp, and the MST did not differentiate 235 pairs of serotypes (Table 2), resulting in an expected specificity of 15.2% for serotyping and 23.9% for serogrouping. These results confirmed XhoII as the enzyme of choice for further analysis.

**Table 2:** Statistical analyses for identification of the most discriminating endonuclease for molecular serotyping.

Enzyme	Number of indistinguishable pairs of serotypes	Specificity for serotyping	Specificity for serogrouping	Median pairwise distances between *cps*-RFLP patterns	Mean pairwise distance between *cps*-RFLP patterns ± Standard Deviation	Median significantly different when compared to XhoII (p < 0.05)
BslFI	17	66.3%	85.8%	29.6	31.6 ± 15.7	Yes
Eco57MI	23	56.5%	89.1%	30.5	32.0 ± 15.2	Yes
HindII	27	54.3%	75%	29.8	31.8 ± 16.0	Yes
HinfI	235	15.2%	23.9%	8.6	10.0 ± 6.4	Yes
StyI	24	63%	76%	32.1	34.6 ± 18.4	No
XhoII	11	76%	100%	32	34.5 ± 17.8	-

### Experimental *cps *RFLP with XhoII

**Figure 2:**
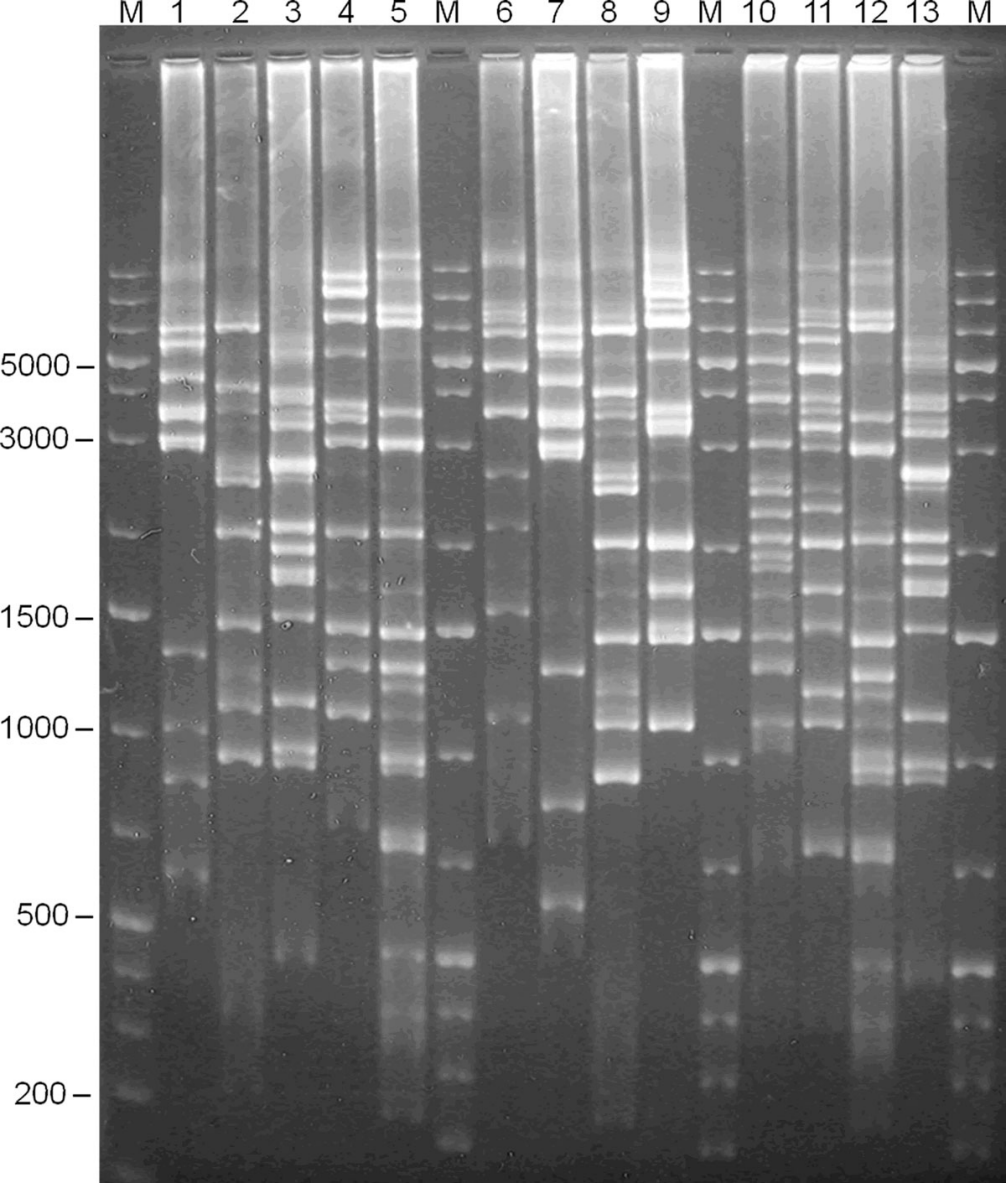
***cps*-RFLP experimental patterns of serotypes 29, 9N, 19F, 6C, 19A, 29, 6A, 19F, 18B, 6B**. Experimental *cps*-RFLP patterns in agarose gel (1.5%). Lanes: M = molecular weight marker; 1 = 79/11-HEM (serotype 29); 2 = 103/11-LCR (serotype 6A); 3 = 149/11-LCR (serotype 9N); 4 = 24/12-LCR (serotype 19F); 5 = 387/11-LCR (serotype 6C); 6 = 124/11-LCR (serotype 19A); 7 = 79/11-HEM (serotype 29); 8 = 103/11-LCR (serotype 6A); 9 = ATCC49619 (serotype 19F); 10 = 240/11-LCR (serotype 18B); 11 = 143/11-LCR (serotype 6B); 12 = 387/11-LCR (serotype 6C); 13 = 149/11-LCR (serotype 9N). Fragment sizes are in base pairs.

PCR amplicons with 17 to 26 kbp were obtained for 43 pneumococci isolates. After digestion with XhoII, 31 distinct patterns were obtained for 29 serotypes represented in this strains collection (the 19F strain produced three different patterns). As expected, the *in vitro cps*-RFLP patterns had four to 17 fragments ranging from 290 to 4,273 bp (Figure 2). To our great dismay, we did not succeed to PCR amplify the *cps *regions of two isolates of serotype 3 and one of serotype 22F after various attempts. This is surprising since Batt and cols. [19] successfully amplified the *cps *region of the reference strains of these two serotypes using the same primers.

Reproducibility of the *cps*-RFLP patterns was confirmed in triplicate assays of 12 isolates of different serotypes randomly chosen. The maximum inter-gel variation in band sizing was 4.33% to the lower size range (0.25 - 0.5 kbp) and 2.23% to the upper size range (0.5 - 4.3 kbp). The intra-gel variation was 1.99% to the lower size range and 1.74% to the upper size range. These limits were lower than the default values of MST, which correspond to the maximal error tolerated in band sizing varying linearly from 7.0% at 0.5 kbp to 3.5% at 4 kbp. Thus, the default parameterization of the MST was maintained.

### Comparison of the *in vitro *and *in silico cps*-RFLP patterns

**Figure 3:**
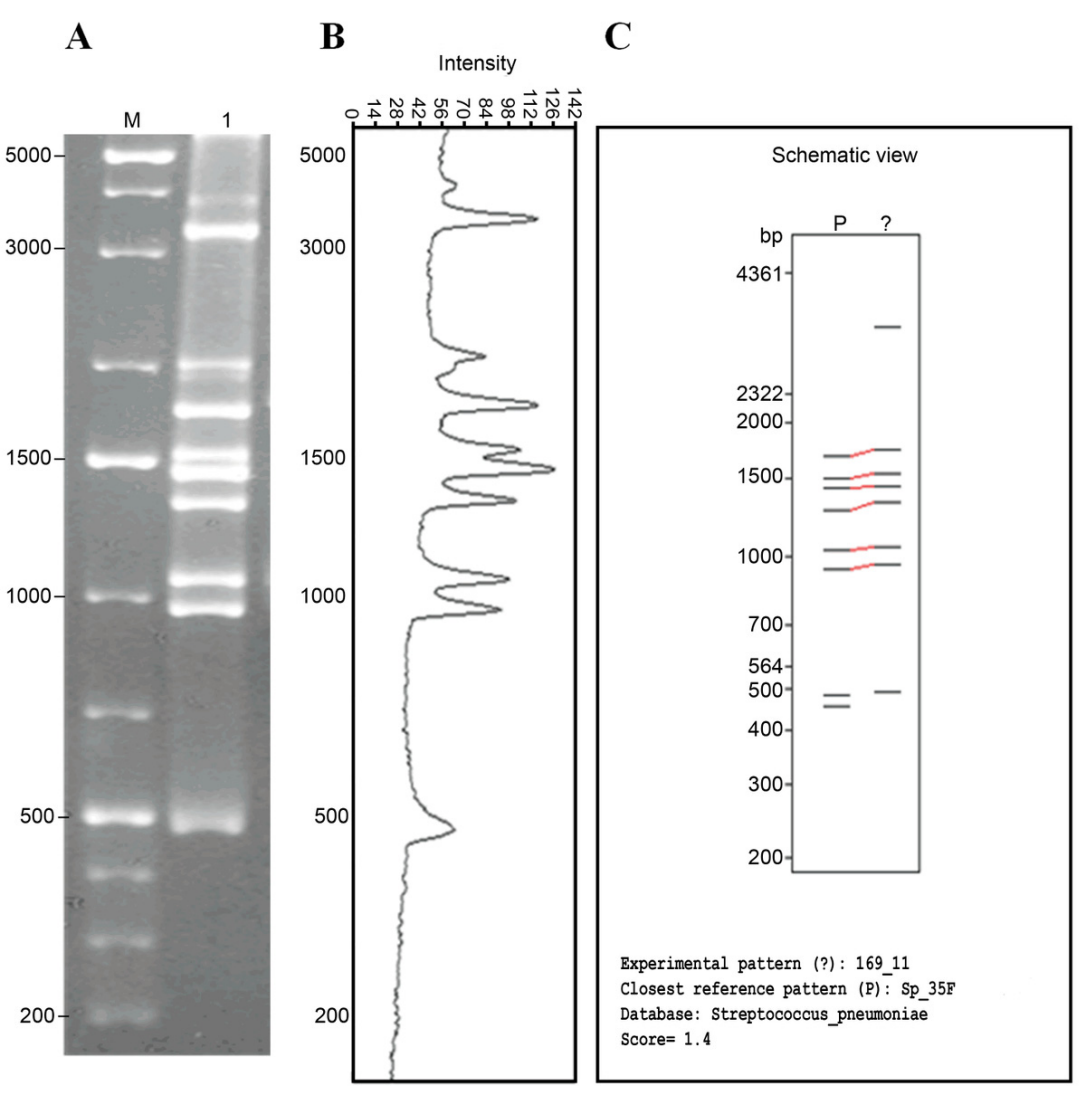
**Correlation between experimentally generated and *in silico *predicted *cps*-RFLP patterns of serotype 35F**. (**A**) Experimental *cps*-RFLP pattern of a strain of serotype 35F in agarose gel (1.5%). Lanes: M = molecular weight marker; 1 = *cps*-RFLP pattern of clinical isolate 169/11-HEM 35F serotype. (**B**) Fragments sizing using the GelAnalyzer software. (**C**) Output of MST showing the schematic representation of the *cps*-RFLP pattern obtained *in vitro *aligned with the closest reference pattern in the database. Fragment sizes are in base pairs.

**Figure 4:**
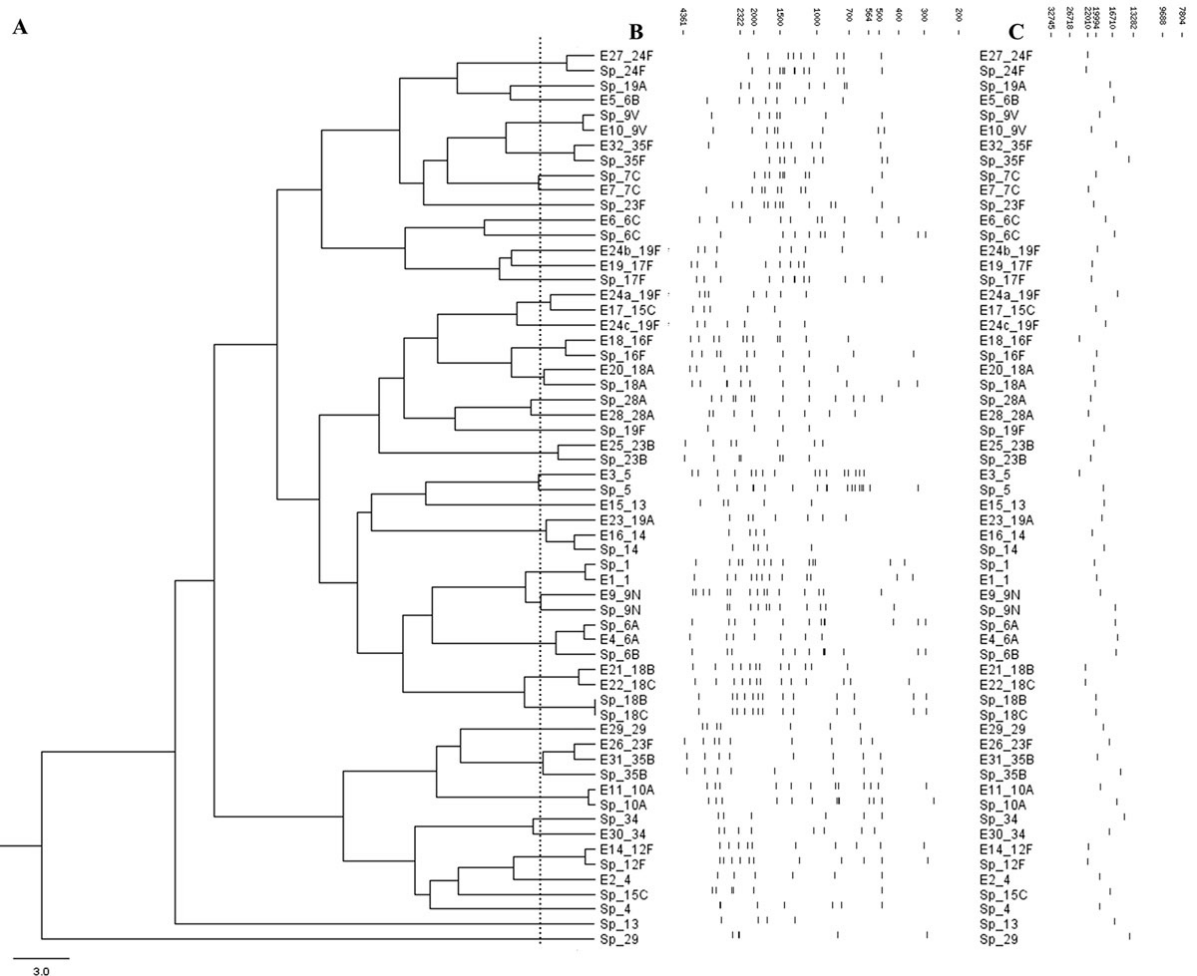
**Clustering the experimental and *in silico cps*-RFLP patterns**. (**A**) Dendrogram showing the results of clustering 31 experimental *cps*-RFLP patterns (E_*) and the *in silico *patterns (Sp_*) of the corresponding serotypes. The dashed line represents the distance threshold under which patterns are indistinguishable by MST software; (**B**) Schematic representation of the *cps*-RFLP patterns; and (**C**) their respective *cps *amplicons. Fragment sizes are in base pairs.

Forty-two clinical isolates of 29 serotypes yielded *in vitro cps*-RFLP patterns, and 11 of these serotypes matched their respective *in silico *patterns. The vast majority of pairs of *in silico *and *in vitro *patterns of the same serotype were similar (Figure 3), as expected, even when the score of the MST alignment was greater than the threshold of 3.0 and the alignments were not considered to be a match. Only eight pairs of patterns were markedly unrelated (Figure 4).

The MST reference database was loaded with the 107 *in silico cps*-RFLP patterns and with 19 *in vitro *patterns that did not match any previous *in silico *pattern under the threshold of similarity score. Altogether, these *cps*-RFLP patterns represent 92 known pneumococci serotypes.

## Discussion

The molecular serotyping method presented herein, *cps*-RFLP with endonuclease XhoII, assesses polymorphisms in the *cps *region of *Streptococcus pneumoniae *allowing serotype identification. In this work, we combined *in silico *and *in vitro *approaches to produce a database of serotype-specific *cps*-RFLP fingerprints that accommodates the genetic diversity within the *cps *locus of 92 known pneumococci serotypes. The database was integrated to MST producing the largest freely accessible dataset of restriction patterns of the *cps *loci of *S. pneumoniae *isolates from different geographical origins.

The Quellung reaction [7,21]with antisera is still the gold-standard for pneumococcal serotyping. However, sera cross-reactivity have already been reported to at least 13 serotype pairs (1/7C, 7A/7C, 7C/7F, 9A/9V, 9N/9V, 9L/9N, 10A/10B, 10A/33, 18B/18C, 18B/18F, 19A/19F, 42/35C and 48/45) [22]. From these, only the pairs 9A/9V, 9L/9N and 18B/18C, could not be distinguished by *cps*-RFLP with XhoII during the *in silico *analysis. They belong to the same serogroups and have very similar *cps *sequences [20].

The *cps*-RFLP patterns were obtained, *in vitro*, using the endonuclease XhoII, for a set of clinical isolates of a well-defined geographical region representing 29 serotypes. Eleven of these serotypes matched their respective predicted patterns in the *in silico *database (37.9%). The large majority of the *cps*-RFLP patterns obtained *in vitro *and their correspondent patterns predicted *in silico *were highly similar (Figure 4). The slight differences observed might be in part explained by the fact that the primers used in the present work [19] are different from those used by Bentley and cols. [9] who sequenced most of the pneumococcal *cps *regions available in Genbank. In fact, we have failed to amplify the *cps *locus using the primers published by Bentley and cols. as reported by others [23]. Some minor differences can also be explained by incomplete digestion, inaccurate fragments sizing, or by co-migration of fragments with very similar molecular weights. The problem of incomplete digestion can be minimized by using an internal control in each experiment (a strain with previously stablished *cps*-RFLP pattern). However, it is worth noting that, in our reproducibility assays, described in the Results section, all *cps*-RFLP patterns obtained for a same strain in different assays were highly similar, and the slight intra- and inter-gel variations could easily be handled by the algorithm of MST. Finally, it is possible that variant patterns for the same serotype may reflect real polymorphisms caused by silent point mutations, insertions, or deletions in the *cps *region that do not alter the CPS antigenic structure. Three different strains of serotype 19F produced three different *cps*-RFLP patterns *in vitro*, most probably due to silent polymorphisms in the XhoII sites in the *cps *region that did not alter the CPS antigenic structure. Similar results had been previously reported in *S. pneumoniae*[16,19], *Shigella *[10], and *E. coli *[11]. Therefore, in order to accommodate this variability, the database of *cps*-RFLP patterns with XhoII was complemented with *in vitro *patterns that did not match those predicted *in silico*.

Molecular serotyping methods based on polymorphisms of the *cps *region of *S. pneumoniae *have been proposed before. Batt and cols. [19] used HinfI to digest the PCR-amplified *cps *regions of 81 epidemiologically unrelated strains representing 46 different serotypes. The patterns obtained were loaded to a database. Afterwards, those authors tested their method against an independent set of 73 isolates from their regional collection, and 43 matched patterns in the database (58.9%). However, it is worth to note that the observed specificity of serotyping methods may be biased by the fact that any single strain collection is differently enriched by serotypes circulating in the geographic regions where samples were collected. Accordingly, our *in silico *simulation demonstrated that *cps*-RFLP with HinfI would have only 15.2% specificity when the 92 known serotypes are considered (Table 2). Additionally, all serotype pairs that were indistinguishable with XhoII could not be differentiated with HinfI. Therefore, the use of endonuclease XhoII significantly increased the specificity of *cps*-RFLP method.

Molecular serotyping methods based on multiplex PCR and microarrays have also been proposed. Yun and cols. [24] developed a multiplex PCR assay to cover the pneumococcal serotypes prevalent in Korea, and Jourdain and cols. [25] designed another version targeting pneumococci epidemiologically relevant in Belgium. These methods require up to eight sequential PCR reactions and amplicon detection steps, and are not readily portable to other geographic regions where pneumococci have different population structures. Alternatively, Raymond et al. [26] described a microarray-based assay to identify *S. pneumoniae *serotypes or serogroups. Assessing 12 polymorphisms located in the capsular operon these authors identified 22 serotypes and assigned 24 other serotypes to a subgroup of serotypes. Another research group developed a microarray incorporating oligonucleotide probes for all known capsular polysaccharide synthesis genes. This array failed to identify only two serotypes in a panel of 91 reference strains representing 91 serotypes [27]. However, further studies with clinical strains from different geographic regions, which can have polymorphisms that are not represented in the microarray, are needed to evaluate the portability of this method. Although promising, microarrays-based molecular serotyping is expensive, and requires statistical analysis of the array intensity data, rendering it unsuitable to be used by researchers in the field of *S. pneumoniae *who are unfamiliar with statistics and bioinformatics. Contrarily, *cps*-RFLP circumvents the main drawbacks of multiplex PCR and microarrays-based techniques, while still achieving 76% of expected specificity for serotyping and 100% for serogrouping. Moreover, *cps*-RFLP performed well in a panel of clinical samples representative of the pneumococci population prevalent in Brazil. Specific PCR assays can be developed with primers designed to detect genes, or gene regions specific of the few serotypes unidentified by *cps*-RFLP. Finally, it is worth note that *cps*-RFLP allows the detection of new serotypes, whose *cps*-RFLP patterns can be added to the database of MST.

The *cps*-RFLP with XhoII could be further improved with a faster DNA purification step. We have also tested the Wizard Genomic DNA Purification Kit (Promega) for long genomic DNA extraction. However, contrarily to previous reports [19,28], the DNA fragments were often degraded and unsuitable for long-extension PCR amplification (data not shown). We have also compared gel electrophoresis and Agilent 2100 Bioanalyzer system (Agilent Technologies, Palo Alto, CA) for separation of fragments in the informative size range of 250 to 4300 bp. For this purpose we used the Agilent DNA 7500 Kit, which covers the size range from 100 to 7500 bp. Bioanalyzer did not perform well with this kit in the size range between 2000 and 4300 bp, where most fragments are concentrated in the *cps*-RFLP patterns (data not shown). Aligned with the main purpose of the present work, which is to provide regional reference laboratories with a simple, low cost, and reliable molecular serotyping method, we cannot recommend Bioanalyzer for the separation of restriction fragments in *cps*-RFLP method until a new kit designed to work in the informative range of fragments sizes is available. Finally, in order to accommodate the genetic variability of the pneumococci *cps *loci, the database of *cps*-RFLP patterns will be progressively expanded to include new variant *in vitro *patterns whenever necessary.

## Conclusions

The *cps*-RFLP method with XhoII as endonuclease and MST for computer-assisted identification of patterns obtained *in vitro *clearly distinguished the large majority of known pneumococcal serotypes. It thus represents a suitable alternative to the Quellung reaction, particularly for small local laboratories that usually only collect the bacterial isolates and send them to reference laboratories to be serotyped. Another advantage of *cps*-RFLP, when compared to other molecular serotyping techniques, is to allow the identification of the capsular ancestry of isolates rendered nonencapsulated due to Single Nucleotide Polimorphisms [29], or even larger indels. When the distance threshold is set to 3.0, the algorithm of MST can handle small variations in fragments sizes and even missing or unexpected fragments in the *cps*-RFLP patterns. The complete database of *cps*-RFLP patterns obtained with XhoII is freely accessible via MST website (http://www.cebio.org/mst), allowing surveillance of local pneumococcal diversity by researchers from any laboratory minimally equipped for Molecular Biology. This may represent a relevant contribution to real time detection of changes in regional pneumococcal population structure in response to recently introduced mass immunization programs.

## List of abbreviations

CPS - Pneumococcal capsular polysaccharide; MST - Molecular Serotyping Tool; PCV10 - 10-valent pneumococcal conjugate vaccine; RFLP - Restriction fragment length polymorphism; UPGMA - Unweighted Pair Group Method with Arithmetic Mean.

## Competing interests

The authors declare that they have no competing interests.

## Authors' contributions

DRAC carried out the microbiological analysis, contributed to the statistical and bioinformatical analysis, and drafted the manuscript.

FSP contributed to the bioinformatical analysis, and drafted the manuscript.

ACV contributed to data analysis, and drafted the manuscript.

MAAO contributed to the microbiological analysis and drafted the manuscript.

RSC conceived and coordinated this study, contributed to the bioinformatical, statistical and microbiological analysis, and wrote the manuscript.

All authors read and approved the final manuscript before submission.

## Supplementary Material

Additional file 1 This table contains the Genbank accession numbers of the *cps *sequences used in this work. Click here for file

## Declarations

Costs for article publication came from FIOCRUZ.

This article has been published as part of *BMC Genomics *Volume 16 Supplement 5, 2015: Proceedings of the 10th International Conference of the Brazilian Association for Bioinformatics and Computational Biology (X-Meeting 2014). The full contents of the supplement are available online at http://www.biomedcentral.com/bmcgenomics/supplements/16/S5.
