# Supplementary material for: Revisitingmolecular serotyping of Streptococcus pneumoniae
Source: BMC Genomics. 2015 May 26;16(Suppl 5):S1. doi: 10.1186/1471-2164-16-S5-S1 (PMC4460616; doi:10.1186/1471-2164-16-S5-S1)
Supplement: Additional file 1 — This table contains the Genbank accession numbers of the cps sequences used in this work. [file 1471-2164-16-S5-S1-S1.docx]

**Table S1 – Accession numbers of all 107 sequences of *cps* loci representing 92 serotypes downloaded from GenBank and their respective serotypes.**

| **Sequences** | **Acession Number ^a^** | **Serotype** |
| --- | --- | --- |
| 1 | CR931632 | 1 |
| 2 | CR931633 | 2 |
| 3 | CR931634 | 3 |
| 4 | CR931635 | 4 |
| 5 | CR931636 | 5` |
| 6 | CR931637 | 5 |
| 7 | CR931638 | 6A |
| 8 | CR931639 | 6B |
| 9 | CR931640 | 7A |
| 10 | CR931641 | 7B |
| 11 | CR931642 | 7C |
| 12 | CR931643 | 7F |
| 13 | CR931644 | 8 |
| 14 | CR931645 | 9A |
| 15 | CR931646 | 9L |
| 16 | CR931647 | 9N |
| 17 | CR931648 | 9V |
| 18 | CR931649 | 10A |
| 19 | CR931650 | 10B |
| 20 | CR931651 | 10C |
| 21 | CR931652 | 10F |
| 22 | CR931653 | 11A |
| 23 | CR931654 | 11B |
| 24 | CR931655 | 11C |
| 25 | CR931656 | 11D |
| 26 | CR931657 | 11F |
| 27 | CR931658 | 12A |
| 28 | CR931659 | 12B |
| 29 | CR931660 | 12F |
| 30 | CR931661 | 13 |
| 31 | CR931662 | 14 |
| 32 | CR931663 | 15A |
| 33 | CR931664 | 15B |
| 34 | CR931665 | 15C |
| 35 | CR931666 | 15F |
| 36 | CR931667 | 16A |
| 37 | CR931668 | 16F |
| 38 | CR931669 | 17A |
| 39 | CR931670 | 17F |
| 40 | CR931671 | 18A |
| 41 | CR931672 | 18B |
| 42 | CR931673 | 18C |
| 43 | CR931674 | 18F |
| 44 | CR931675 | 19A |
| 45 | CR931676 | 19B |
| 46 | CR931677 | 19C |
| 47 | CR931678 | 19F |
| 48 | CR931679 | 20 |
| 49 | CR931680 | 21 |
| 50 | CR931681 | 22A |
| 51 | CR931682 | 22F |
| 52 | CR931683 | 23A |
| 53 | CR931684 | 23B |
| 54 | CR931685 | 23F |
| 55 | CR931686 | 24A |
| 56 | CR931687 | 24B |
| 57 | CR931688 | 24F |
| 58 | CR931689 | 25A |
| 59 | CR931690 | 25F |
| 60 | CR931691 | 27 |
| 61 | CR931692 | 28A |
| 62 | CR931693 | 28F |
| 63 | CR931694 | 29 |
| 64 | CR931695 | 31 |
| 65 | CR931696 | 32A |
| 66 | CR931697 | 32F |
| 67 | CR931698 | 33A |
| 68 | CR931699 | 33B |
| 69 | CR931700 | 33C |
| 70 | CR931701 | 33d |
| 71 | CR931702 | 33F |
| 72 | CR931703 | 34 |
| 73 | CR931704 | 35A |
| 74 | CR931705 | 35B |
| 75 | CR931706 | 35C |
| 76 | CR931707 | 35F |
| 77 | CR931708 | 36 |
| 78 | CR931709 | 37 |
| 79 | CR931710 | 38 |
| 80 | CR931711 | 39 |
| 81 | CR931712 | 40 |
| 82 | CR931713 | 41A |
| 83 | CR931714 | 41F |
| 84 | CR931715 | 42 |
| 85 | CR931716 | 43 |
| 86 | CR931717 | 44 |
| 87 | CR931718 | 45 |
| 88 | CR931719 | 46 |
| 89 | CR931720 | 47A |
| 90 | CR931721 | 47F |
| 91 | CR931722 | 48 |
| 92 | EF538714 | 6C |
| 93 | HM171374 | 6D |
| 94 | Z83335 | 1 |
| 95 | AF026471 | 2 |
| 96 | AE005672 | 4 |
| 97 | AF316639 | 4 |
| 98 | AF246897 | 6B |
| 99 | AF316640 | 6B |
| 100 | AJ239004 | 8 |
| 101 | AF402095 | 9V |
| 102 | X85787 | 14 |
| 103 | AF094575 | 19A |
| 104 | U09239 | 19F |
| 105 | AF030373 | 23F |
| 106 | AF057294 | 23F |
| 107 | AJ006986 | 33F |

**^a^** References: sequences 1 to 91 [9]; sequence 92 [17]; sequence 93 [18]; and sequences 94 to 107 [16].
